# Observation of enhanced superconductivity in the vicinity of Ar-induced nano-cavities in Pb(111)

**DOI:** 10.1038/s41598-017-12505-1

**Published:** 2017-09-22

**Authors:** Sang Yong Song, Jungpil Seo

**Affiliations:** Department of Emerging Materials Science, DGIST, 333 Techno-Jungang-daero, Hyeonpung-Myun, Dalseong-Gun, Daegu, 42988 Korea

## Abstract

Local variations of superconductivity have been studied using scanning tunneling microscopy around nano-cavities formed by Ar ions embedded in Pb(111). Various factors including the density of states at Fermi energy, electron–phonon couplings, and quantum well states, which are known to affect superconductivity, have been examined. We show that the superconductivity is enhanced near the nano-cavities and propose that quantum effects such as quantum confinement, proximity effect and multi-gap effect are possibly involved in determining the superconducting gap of this system. These results have important implications for the characterization and understanding of superconductivity at a nanometer scale.

## Introduction

Superconductivity of Pb film is particularly interesting because its superconducting transition temperature (*T*c) can be modified by the coupling to the substrate and/or quantum effects by the reduced dimension^1–14^. The Bardeen–Cooper–Schrieffer (BCS) theory predicts that the *T*c value of s-wave superconductors is strongly influenced by the density of states (DOS) at Fermi energy (*E*_F_)^15^. While Guo *et al*. reported the quantum confinement effect in superconductivity of Pb films^5^, Eom *et al*. microscopically correlated the quantum confinement effect with the *Tc* oscillations^2^.

Various quantum phenomena occur in the vicinity of Ar-induced nano-cavities (AICs) when Ar gas is trapped in Pb substrate^16,17^. Quantum well states (QWSs) arise from parallel interfaces of vacuum-Pb-AIC. Unlike the Pb films grown on Si, the AICs in Pb have weak interface bonding in the vicinity and provide almost free-standing Pb films to study, which minimizes the substrate effect in superconductivity. Moreover, Muller *et al*. reported that lateral confinement is caused by reflections from the laterally open boundary of AICs^17^.

Pb is known as a strong-coupling s-wave superconductor. It is described adequately by the Migdal–Eliashberg theory, which highlights the role of the electron–phonon couplings depending on energy in superconductivity^18^. Furthermore, the Fermi surface of Pb contains two separate Fermi sheets (FSs) that consist of a spherical inner FS and a tubular outer FS. Each FS leads to a different superconducting gap of Δ_1_ and Δ_2_^19–22^. The multi-gap feature together with the quantum confinements and strong electron–phonon couplings make Pb films formed on AICs complicated and interesting for the study of superconductivity.

In this paper, we studied the changes of superconductivity in the vicinity of the AICs using scanning tunneling microscopy and spectroscopy (STM/STS) at 2.7 K. To minimize the thermal broadening effect in maximizing the energy resolution of the spectrum, we used a superconducting tip coated with Pb (see Fig. S1 in the Supplementary Information)^23^. By using this tip, we could obtain the energy resolution of the spectrum down to 30 µeV in the experiment. In the experiment, we unambiguously observed the superconductivity is enhanced near the Pb films on AICs. Such variation of superconducting gap at a nanometer scale has been sought in Pb films grown on Si, but not directly observed^6,24^. We show the enhanced superconductivity in this system is not directly correlated with the change of the electron-phonon couplings.

## Results

### [Quantum Confinement Effect and AICs]

**Figure 1:**
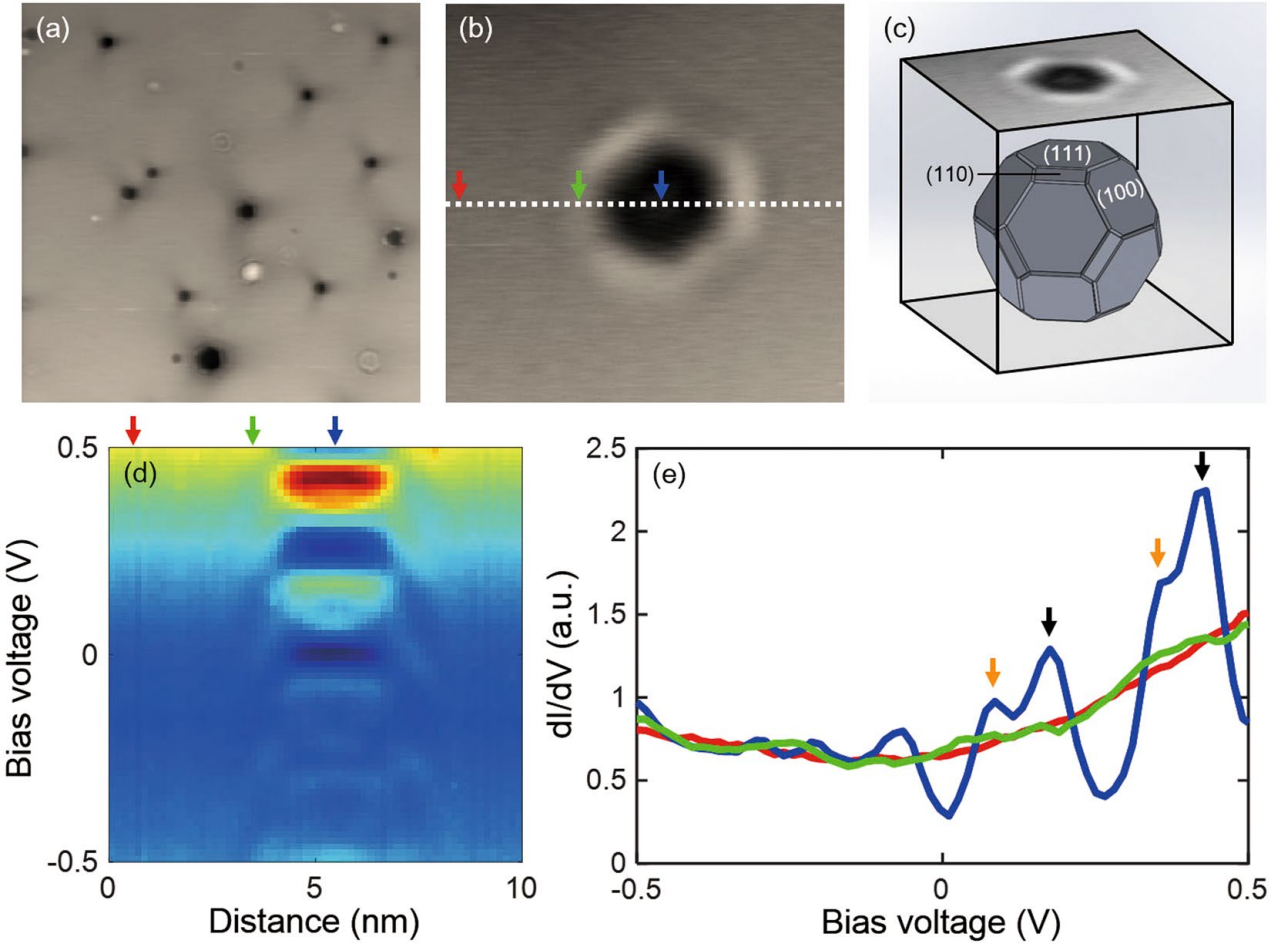
Ar-induced nano-cavities (AICs) in Pb(111). (**a**) 100 nm × 100 nm image of the Ar surface bubbles (SBs) on Pb(111) (*V*_bias_ = 50 mV, *I* = 50 pA). (**b**) Zoomed-in image of an SB with the diameter of ~4 nm (*V*_bias_ = 50 mV, *I* = 50 pA). (**c**) The schematic image of an AIC in Pb(111). The growth of the cavity follows the Wulff construction in Pb(111). (**d**) The spatial dependence of quantum well state (QWS) spectra measured along the white dotted line in (**b**). (**e**) Differential conductance (*dI*/*dV*) spectrum measured on bulk (red arrow), SB side (green arrow), and SB center (blue arrow) (*V*_bias_ = 0.5 V, *I* = 100 pA). The main peaks (black arrows) and sub-peaks (orange arrows) originate from vertical and lateral QWS, respectively.

During the Ar sputtering and annealing process (see Method), Ar ions are embedded inside the Pb crystal and their equilibrium shapes follow the Wulff construction in the crystal environment (Fig. 1b and c)^16^. They appear as surface bubbles (SBs) on the Pb(111) when measured by STM. Figure 1a shows the STM image of various SBs with diameters ranging from 2 nm to 10 nm on Pb(111). In this system, Pb films are formed by sandwiching between the vacuum and the AICs. The quasiparticles are confined vertically in the sandwiched Pb films and laterally by open boundary reflections near the SBs. Figure 1d shows spatial variations of the DOS for quasiparticles near an SB when spectra are measured along the white dotted line in Fig. 1b. At the SB center (blue arrow in Fig. 1b and d), QWS peaks are clearly seen (blue line in Fig. 1e). The peaks are composed of main peaks (black arrows) and sub-peaks (orange arrows), which arise from vertical and lateral confinement, respectively (see Fig. S2 in the Supplementary Information). At the center of SBs, the DOS near *E*_F_ is changed by QWS as a function of the AIC depth, which allows us to examine the relationship between superconductivity and the DOS near *E*_F_. The spectrum at the SB side (green arrow in Fig. 1b and d, enhanced superconducting gap area; see later) is similar to that at Pb bulk (red arrow in Fig. 1b and d).

### [Variation of Superconductivity near SBs]

**Figure 2:**
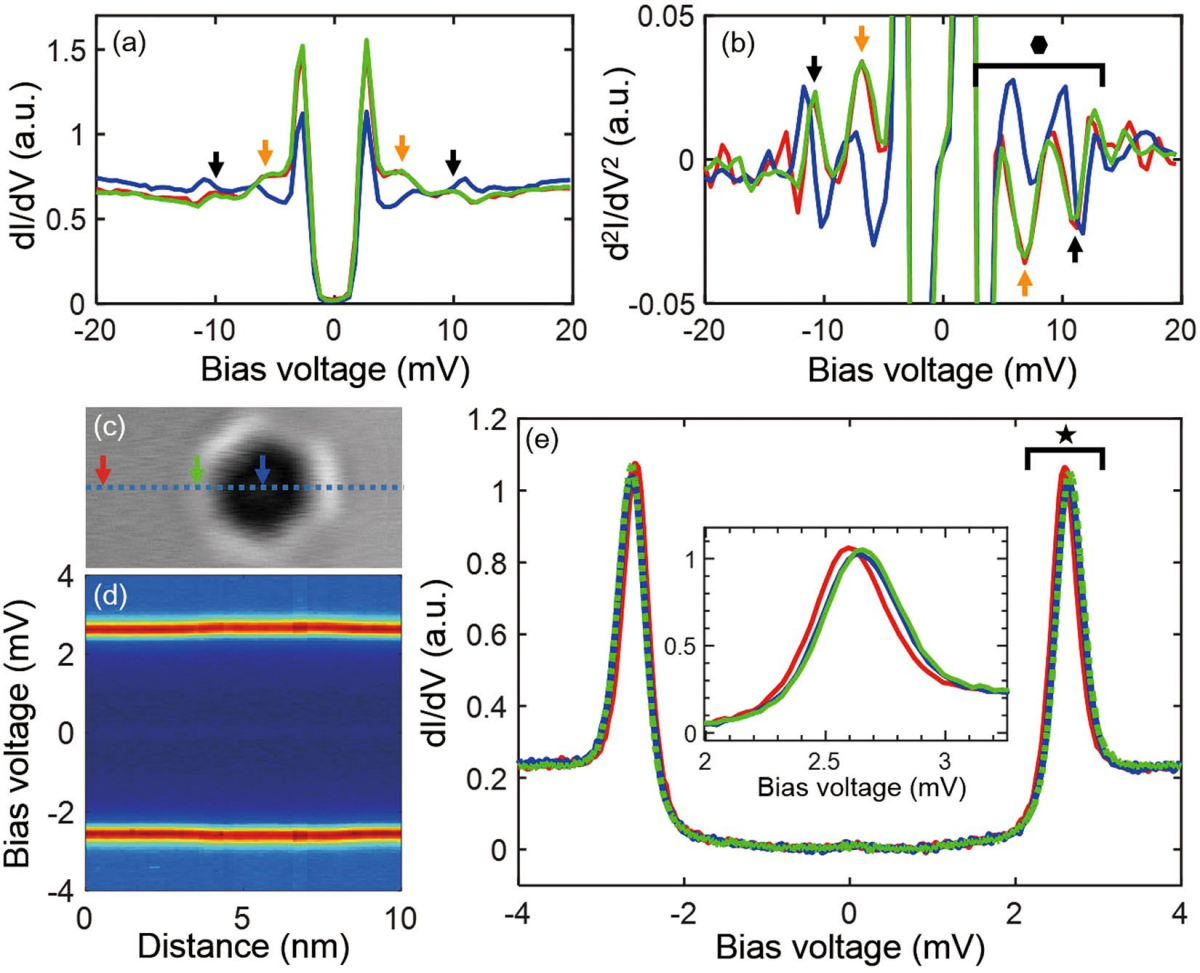
Electron–phonon coupling peaks and superconducting gap. (**a**) *dI*/*dV* spectra measured on bulk (red arrow in (**c**)), SB side (green arrow in (**c**)), and SB center (blue arrow in (**c**)). Four humps appear beyond the superconducting gap (black and orange arrows). (**b**) The derivative of the *dI*/*dV* spectra (*d* ^2^ *I*/*dV* ^2^) obtained in (**a**). The peaks appear at ±6.386 ± 0.05 mV (orange arrows) and ±11.23 ± 0.05 mV (black arrows). These peaks are related to the electron–electron interactions dressed by the phonon in superconductivity. (**c**) STM topography image of an SB. (**d**) The spatial dependence of −4 mV to 4 mV spectra measured along the white dotted line in (**c**). (**e**) *dI*/*dV* spectra measured on bulk (red line), SB side (green line), and SB center (blue line). The superconducting gap increases as much as 60 µV at the SB center and SB side (inset).

**Figure 3:**
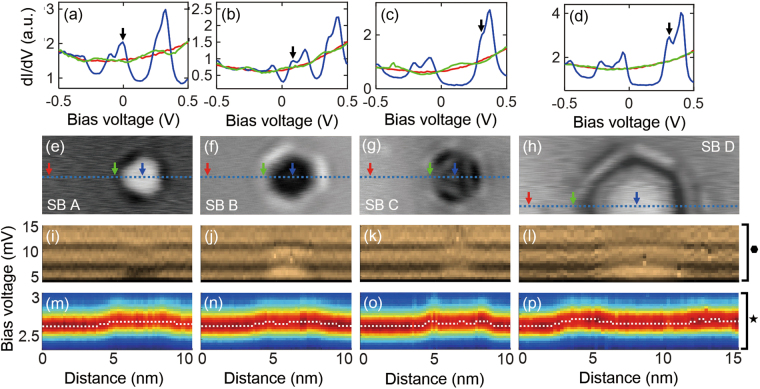
Density of states (DOS) at Fermi energy (*E*_F_), spatial variation of phonon peaks, and superconducting gap in the vicinity of SBs. (**a**–**d**) The DOS of bulk (red line, red arrow in (**e**–**h**)), SB side (green line, green arrow in (**e**–**h**)) and SB center (blue line, blue arrow in (**e**–**h**)) in SBs A, B, C, and D. The black arrow indicates the position of the lowest unoccupied state (LU). (**e**–**h**) SBs A, B, C, and D with the diameter (depth) of approximately 4 nm (34 ML), 4 nm (44 ML), 4 nm (26 ML), and 7.5 nm (25 ML), respectively. (**i**–**l**) The spatial dependence of *d* ^2^ *I*/*dV* ^2^ spectra in the range of 5–15 mV (indicated by the hexagon mark in Fig. 2(b)) across each SB (along the blue dotted line in (e–h)). Two black lines in data correspond to the trace of the phonon peaks (+6.386 ± 0.05 mV and +11.23 ± 0.05 mV at bulk). (**m**–**p**) Spatial dependence of superconducting gap spectra (2.3–3 mV) across each SB (along the blue dotted line in (**e**–**h**)). The superconducting gap is increased in the vicinity of SBs.

Figure 2d shows the spatial dependence of the *dI*/*dV* spectra in the range of −4 mV to 4 mV measured with 30-µV resolution across the center of an SB (blue dotted line in Fig. 2c). From these data, we visually confirm the increase of the superconducting gap near the SB. The superconducting gap increased by 60 µV on both the side and center of this SB (Fig. 2e and inset). In general, we observe that the increase of the superconducting gap at the SB center is equal to or smaller than that at the SB side (Fig. 3m–p).

### [Phonon Peaks near SBs]

The superconductivity of Pb is explained by the Migdal–Eliashberg theory, which predicts that the structure of the electronic DOS is related to the electron–phonon spectrum^18,25,26^. In the experiment, we measured variations of the phonon peaks in the vicinity of SBs using a superconducting tip. In *dI*/*dV* spectra over the range of −20 mV to 20 mV, four humps appeared beyond the superconducting gap (black and orange arrows in Fig. 2a). Considering the derivative of the *dI*/*dV* spectra (*d* ^2^ *I/dV* ^2^), the peak positions and intensities associated with the humps are identified more accurately (Fig. 2b). The peaks are located at ±6.386 ± 0.05 mV (orange arrows) and ±11.23 ± 0.05 mV (black arrows), and they correspond to transverse mode phonon and longitudinal mode phonon peaks, respectively^25^. The phonon peaks at Pb bulk (red line in Fig. 2b) and at the SB side (green line in Fig. 2b) are similar in terms of peak positions and intensities, whereas the phonon peaks at the SB center (blue line in Fig. 2b) show weaker intensity with a position shift of 0.5 mV compared to the bulk and SB side.

Because we used a superconducting tip to resolve the superconducting gap near the SB, the spectra we obtained include the phonon peak contributions from both the tip and sample. To remove the ambiguity, we performed the deconvolution for the spectra in order to obtain intrinsic spectra of the sample (see Fig. S3 in the Supplementary Information)^27^. We find that the phonon peaks in the intrinsic spectra are shifted by a superconducting gap of Pb, but the variation of phonon peaks remains consistently among the SB center, SB side and bulk.

### [Superconductivity versus the DOS at *E*_*F*_]

To investigate the relationship between the DOS at *E*_F_ and the enhanced superconductivity near SBs, we have selected four SBs that show a characteristic QWS at *E*_F_. The diameter of SBs A, B, and C is approximately 4 nm, and that of SB D is approximately 7.5 nm (Fig. 3e–h). The depths of SBs A, B, C, and D are approximately 34, 44, 26, and 25 monolayer (ML; 1 ML ~0.283 nm), respectively (see Fig. S4 in the Supplementary Information). When one of the QWSs is located on *E*_F_, the DOS near *E*_F_ is higher than bulk. This is true for the case of SB A (Fig. 3a). On the other hand, when *E*_F_ is between the QWS, the DOS at *E*_F_ is lower than bulk. This is true for the case of SBs B, C, and D (Fig. 3b–d). Figure 3m–p show the spatial variation of the superconducting gap across SB in the range of 2.3–3.3 mV (this corresponds to the star mark in Fig. 2e). Because higher DOS at *E*_F_ results in the stronger electron–phonon coupling according to the BCS theory, the enhanced gap (60 µV) at the center of SB A (Fig. 3m) is somewhat expected. However, surprisingly, we also observed an increase of the superconducting gap by 60 µV, 30 µV, and 30 µV for SBs B (Fig. 3n), C (Fig. 3o), and D (Fig. 3p), respectively, for which the DOS at *E*_F_ is lower than bulk.

**Figure 4:**
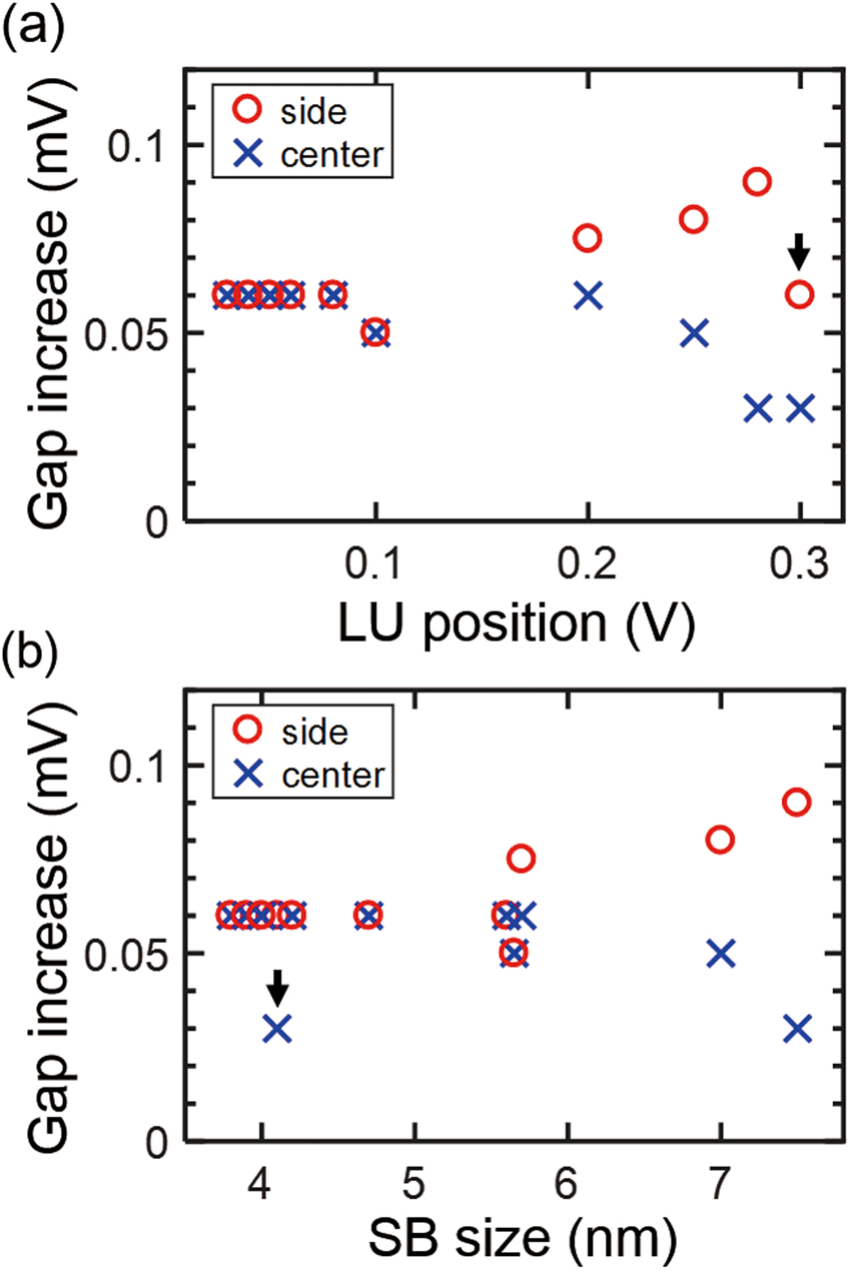
The enhanced superconducting gap as a function of the position of LU and the SB size. (**a**) The increase of the superconducting gap depends on the LU position at the SB center. (**b**) The increase of the superconducting gap is correlated with the SB size at the SB side. The black arrows indicate the counter examples that break the relationship between the gap increase at the SB side and the LU position in (**a**) and between the gap increase at the SB center and the SB size in (**b**).

Comparing the DOS at *E*_F_ among SBs is not very straightforward because many of the SBs are measured by an STM tip with different conditions. Therefore, following the literature (ref^24^.), we exploit the position of the lowest unoccupied QWS (LU) in the SB center to identify the relative magnitude of DOS at *E*_F_. The farther the LU from *E*_F_, the less the DOS is affected by the broadened peak of LU (see Fig. S5 in the Supplementary Information). As a result, as the position of LU deviates from *E*_F_, the DOS decreases at *E*_F_. Figure 4 exhibits how the LU position affects the superconductivity at the SB center (blue cross marks) and at the SB side (red circle marks). At the SB center, the increasing amplitude of the gap size depends on the LU position. Namely, the effect of increasing the superconducting gap is reduced when the DOS at *E*_F_ is lowered at the SB center.

On the other hand, we found a significant exception in estimating the relation between the increase of the superconducting gap at the SB side and the LU position (a black arrow in Fig. 4a). Furthermore, the superconducting gap is obviously increased near the SB side for every case (Fig. 3m–p) although the DOS at *E*_F_ is similar to the bulk (green and red lines in Fig. 3a–d). Our experiment unambiguously shows that the increase of superconductivity at the SB side is not correlated with the DOS at *E*_F_.

### [Superconductivity versus Phonon Peaks]

To know the spatial variation of the phonon peaks, we plotted *d* ^2^ *I*/*dV* ^2^ spectra in the range of 5–15 mV (indicated by the hexagon mark in Fig. 2(b)) across the SB. As shown in Fig. 2b, two phonon peaks appear as dips in the positive bias regime. Therefore, in Fig. 3i–l, two black lines correspond to the trace of the phonon peaks. When the DOS at *E*_F_ in the SB center is higher than that in bulk, the phonon peaks are shifted downward by 0.5 mV (Fig. 3i). Meanwhile, the lower DOS at *E*_F_ compared to bulk causes the phonon peaks to shift upward by 0.5 mV (Fig. 3j–l). Therefore, our experiment revealed that the sign of the peak shift is correlated with the DOS at *E*_F_. However, we could not find the relationship between the phonon peak shift and the enhanced superconducting gap (30–60 µV) at the SB center. This indicates that change of phonon frequency may not necessarily alter the overall electron-phonon coupling strength in superconductivity. The positions of phonon peaks are almost identical to the bulk at the SB side (Fig. 3i–l), indicating that the increase of the superconducting gap (60–90 µV) at the SB side is irrelevant to the phonon peaks.

### [Superconductivity versus the Size of SBs]

For the SBs we measured, we observed that the superconducting gap at the SB side is always larger than that at the SB center. Interestingly, we found that the increase of superconducting gap at the SB side is correlated with the size of an SB (red circle marks in Fig. 4b). For example, SBs of ~4 nm size induced a gap increase of 60 µV (Fig. 3m–o), but SB of ~7.5 nm size induced an increase of 90 µV (Fig. 3p). In contrast, determining the relationship between the increase of the superconducting gap at the SB center and the SB size is not straightforward owing to the exception indicated by the black arrow in Fig. 4b.

## Discussion

In the experiment, we unambiguously observed that the superconducting gap is enhanced near the SBs formed by AICs in Pb(111). Although the DOS at *E*_F_ is barely changed at the SB side compared to Pb bulk, the gap at the SB side is significantly increased by 60–90 µV, which is different from previous reports in which the superconducting gap is correlated with the DOS at *E*_F_^2,5,8,11^. In addition, the electron-phonon couplings, which are characterized by the intensity and location of phonon peaks in *dI* ^2^ */dV* ^2^, are almost identical to each other for the bulk and the SB side in spite of the variation of superconducting gap. This implies that other mechanisms in addition to electron–phonon interactions play a role in the superconductivity of this system.

As mechanisms for explaining the enhanced superconductivity at the SB side, we suggest two possibilities. The first is a multi-gap scenario for Pb films. The Fermi surface of Pb contains two separate Fermi sheets (FSs) that consist of a spherical inner FS and a tubular outer FS. Each FS leads to a different superconducting gap of Δ_1_ and Δ_2_^19–22^. The contribution of Δ_2_ to the superconducting gap is minimized along the [111] direction while the contribution of Δ_1_ does not depend on the crystal direction^22^. In the experiment, we observed the area of enhanced superconducting gap at the SB side increases as the size of AIC (thus the size of the crystalline facets of AIC) increases (see Fig. S6 in the Supplementary Information). Furthermore, we find the enhanced superconductivity follows three-fold rotational symmetry of the facets of AICs (see Fig. S6 in the Supplementary Information). Therefore, it seems that there is a strong correlation between the enhanced superconductivity and the presence of the buried side facets of AICs. If the FSs can be distorted by the side facets of AICs, the contribution from Δ_2_ to the superconducting gap would be increased, which likely results in an increased superconducting gap due to the thermal broadening.

**Figure 5:**
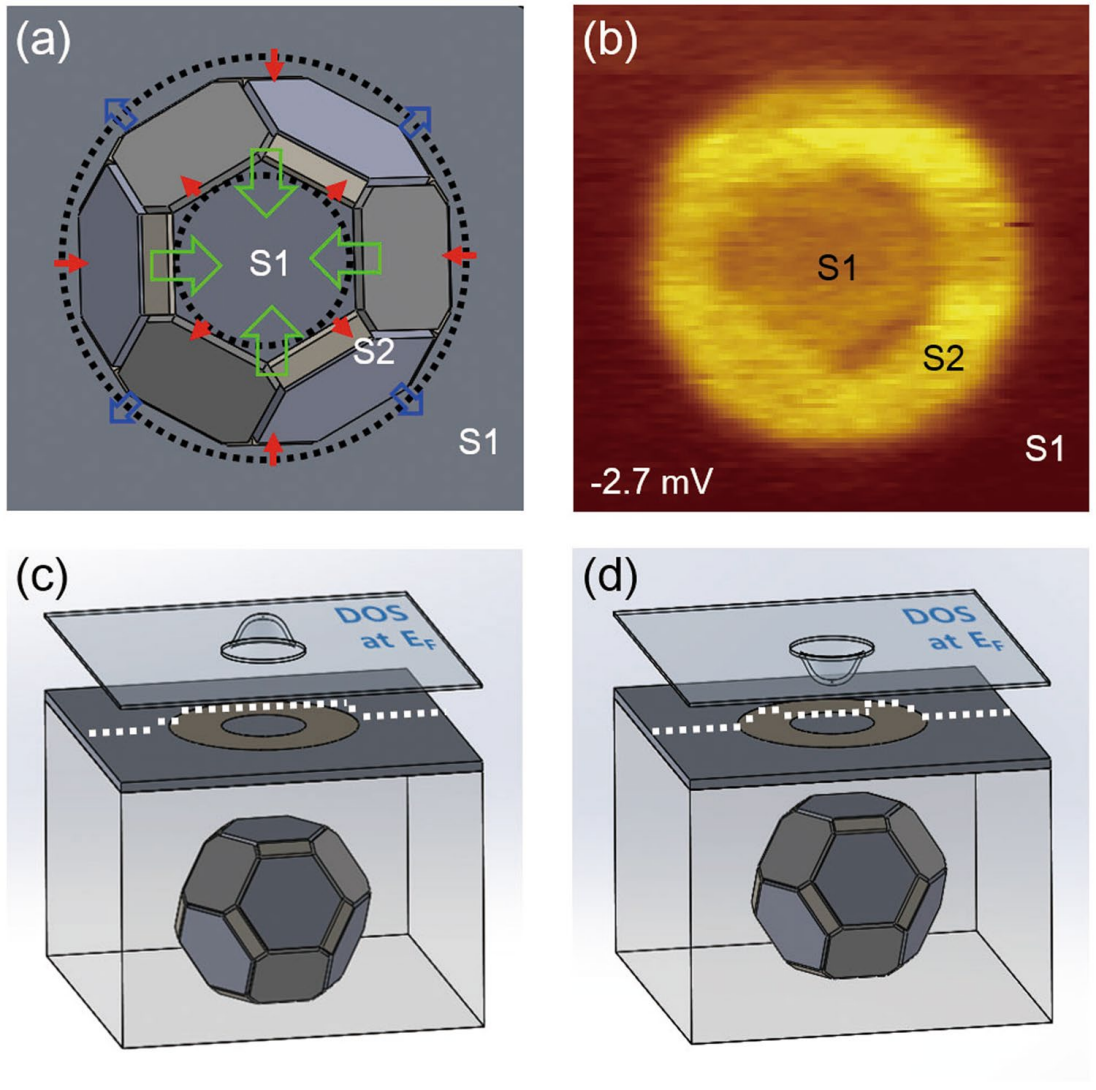
Superconductor–superconductor (S–S) proximity effects and the DOS in the vicinity of AIC. (**a**) Three kinds of proximity effects. The blue arrows, red arrows, and green arrows indicate the S–S proximity, S–S inverse proximity, and S–S proximity for the surrounded junction, respectively. (**b**) The *dI*/*dV* map at −2.7 mV (**c**) When the DOS at *E*_F_ in the SB center is high, the proximity-induced superconductivity from the SB side effectively survives at the SB center (white dotted line). (**d**) When the DOS at *E*_F_ in the SB center is low, the induced superconductivity from the SB side is decreased (white dotted line).

Another scenario is that non-uniformly formed Cooper pairs in space by quantum confinement may affect the superconductivity. Croitoru *et al*. suggested that discrete electron levels such as QWS break the translational invariance of a superconducting system. The superconducting order parameter then depends on the position in space, which spontaneously induces the spatial variation of the superconducting gap^28^. Because the translational symmetry is broken most effectively at the boundary of SB, which is the interface between the QWS on AICs and continuum bulk states, the gap size is prominently affected at the SB side. This agrees with our experiment in which the superconducting gaps are enhanced most significantly at the SB side (Fig. 5b).

The enhanced superconductivity at the SB side can affect the superconducting gap at the SB center through the proximity effects. When a stronger superconductor (SB side) with a finite electron reservoir makes contact with weaker superconductors (bulk and SB center), the gap of the stronger superconductor is weakened by the inverse proximity effect (red arrows in Fig. 5a)^29^, which depends on the size of the reservoir. The size of the electron reservoir at the SB side increases as the size of the SB is increased (see Fig. S6 in the Supplementary Information). Therefore, when the size of the SB is larger, the superconductivity at the SB side is more robust to the inverse proximity effect. This might explain the increase of the gap at the SB side depends on the size of the SB (Fig. 4b).

The relatively weak superconducting region at the SB center is surrounded by the strong superconducting region at the SB side. Because the diameter of the surrounded region is several nanometers, the Andreev reflection converges from all surrounding sides^30,31^, which makes the proximity effect stronger at the SB center than bulk near the SB side (green and blue arrows in Fig. 5a). Figure 5b shows the conductance map measured at a bias voltage of −2.7 mV, which corresponds to the quasi-particle peak of the spectrum at the SB side. The SB region is brighter than bulk owing to the difference of the gap size (see Fig. S7 in the Supplementary Information).

The superconductivity at the SB center is also affected by QWS from vertical and lateral confinement. Figure 4a shows that the superconducting gap is decreased as the DOS at *E*_F_ is lowered, which implies that the proximity effect is diminished at the SB center, due to the lack of the states for Cooper pair tunneling as the DOS at *E*_F_ is decreased. Figure 5c and d show the schematic images of the DOS (blue plane) and variation of the superconducting gap (white dotted line) near AICs.

In conclusion, we have investigated the local variations of superconducting gap near SBs using STM. We observed that the superconducting gap is enhanced at the SB side compared to bulk although both the phonon peaks and the DOS at *E*_F_ remain similarly. Our experiment shows that the superconducting gap at the SB side is related to the size of the SB but not the DOS at *E*_F_. At the SB center, the superconducting gap is correlated with the DOS at *E*_F_ but not with the size of SB. As possible origins of the enhanced superconducting gap at the SB side, we suggest the multi-gap contributions and the Cooper pair modulation at the spatial interface between the QWS and bulk states, which are not both conclusive yet. We also propose the superconducting gap at the SB center is involved with the proximity effect which is affected by the geometry and the DOS at *E*_F_. However, it is unclear how the phonon peaks at the SB center are shifted significantly. Besides, it is also necessary to be explored how the phonon peaks shift in energy depending on the DOS at *E*_F._ Our experimental results are not explained by the electron-phonon coupling theory alone and call for future theoretical works.

## Methods

### STM Measurement

We have carried out the experiment using a home-built STM operating at 2.7 K in the base pressure of 5 × 10^−11^ torr. The AICs in Pb(111) were prepared by 5 cycle processes of 2 kV Ar^+^ sputtering during 10 minutes in Ar pressure of 4.5 × 10^−5^ torr and annealing at 500 K during 12 minutes. We intentionally poked a Pt-Ir tip onto the Pb substrate to make the tip superconducting. The differential conductance (dI/dV) spectra were obtained using a standard lock-in technique with the modulation frequency of 463 Hz and the root-mean-square amplitude of 30 µV~10 mV. To reduce the radio-frequency (RF) noise in the measurement we employed PI filters (PN410-1067-ND, Digikey) for the signal lines and a metal powder filter for the current line.

### Data Availability

The datasets generated during and/or analysed during the current study are available from the corresponding author on reasonable request.

## Electronic supplementary material

Supplementary Information
