# Supplementary material for: Observation of enhanced superconductivity in the vicinity of Ar-induced nano-cavities in Pb(111)
Source: Sci Rep. 2017 Sep 22;7:12177. doi: 10.1038/s41598-017-12505-1 (PMC5610268; doi:10.1038/s41598-017-12505-1)
Supplement: Supplementary file 1 — Supplementary Information [file 41598_2017_12505_MOESM1_ESM.pdf]

Supplementary Information

Observation of enhanced superconductivity in the vicinity of Ar-induced nano-cavities in Pb(111)

Sang Yong Song and Jungpil Seo*

Department of Emerging Materials Science, DGIST, 333 Techno-Jungang-daero, Hyeonpung-Myun, Dalseong-Gun, Daegu 42988, Korea

*Address correspondence to jseo@dgist.ac.kr

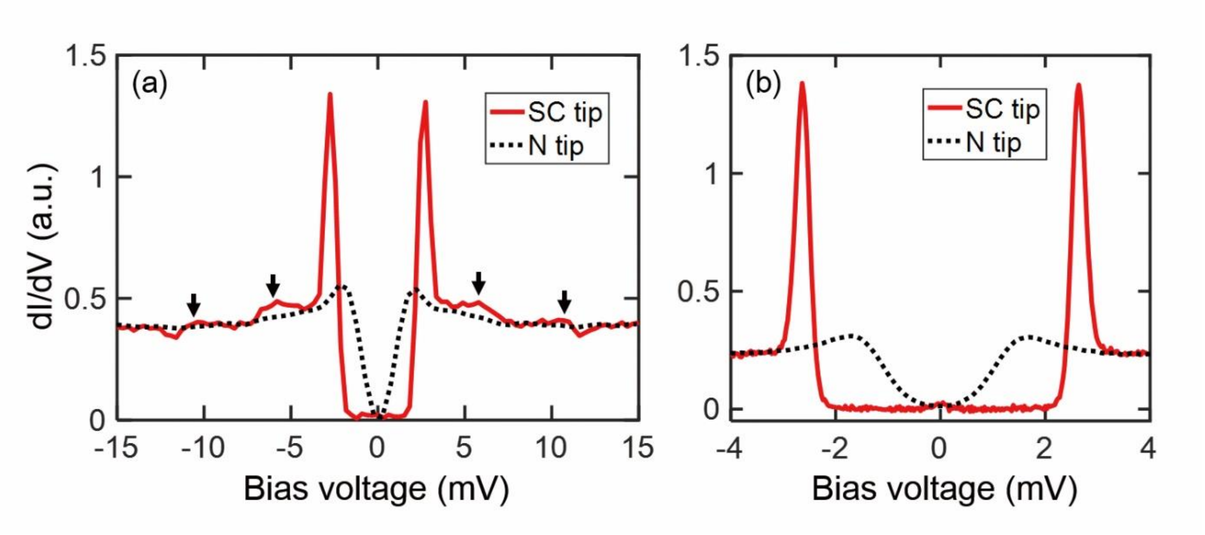

Figure S1. Differential conductance spectra obtained by the superconducting tip and normal tip at 2.7K. (a) The black dotted line and red solid line represent the dI/dV spectra measured on Pb(111) using a normal (N) tip and Pb-coated superconducting (SC) tip, respectively. The humps associated with the electron-phonon coupling are clearly resolved by the SC tip (vertical arrows). (b) The superconducting gap obtained by the N tip (black dotted line) and the SC tip (red solid line). When the SC tip is used, the quasiparticle resonance peaks appear sharp and clear, which allows us to determine the variation of superconducting gap with a high precision.

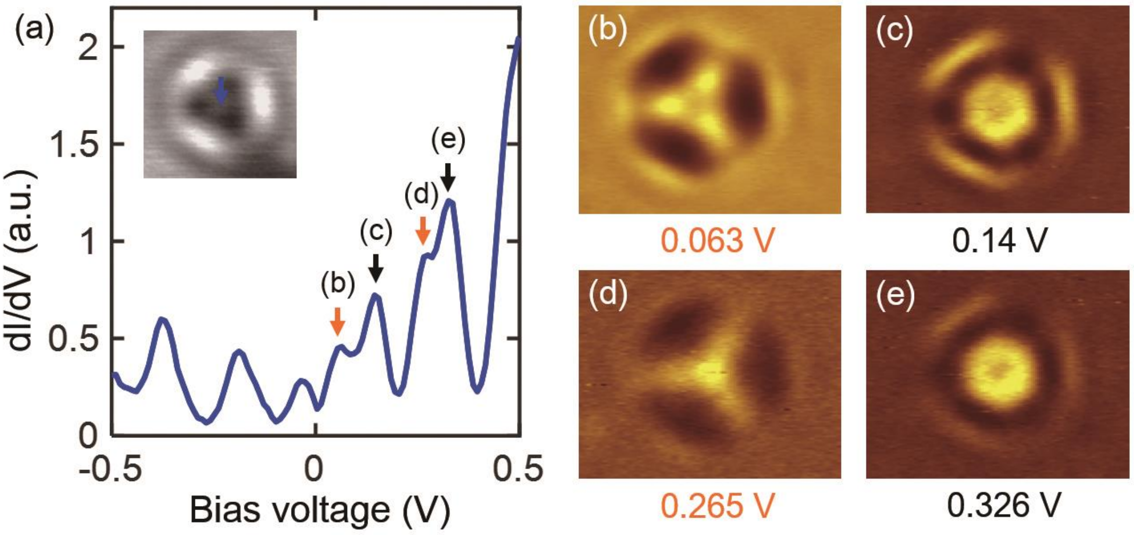

Figure S2. Vertical and lateral confinement on the surface bubble (SB). (a) dI/dV spectra measured on SB center (blue arrow in inset). The main peaks (black arrows) and subpeaks (orange arrows) correspond to quantum well states (QWSs) originate from vertical and lateral confinement, respectively. Inset shows the STM image of an SB of which depth is 57 ML (V_{bias} = 0.05 V, I = 50 pA). The image size is 5 nm × 5 nm. (b–e) The conductance map at the bias voltage of 0.063 V, 0.14 V, 0.265 V, and 0.326 V, respectively. The hexagonal pattern (c) and (e) result from the vertical confinement (black arrows in (a)). The less symmetrical pattern (b) and (d) result from the lateral confinement (orange arrows in (a)) which is influenced by the side facets of the underlying Ar cavity (Fig. 1c in main text).

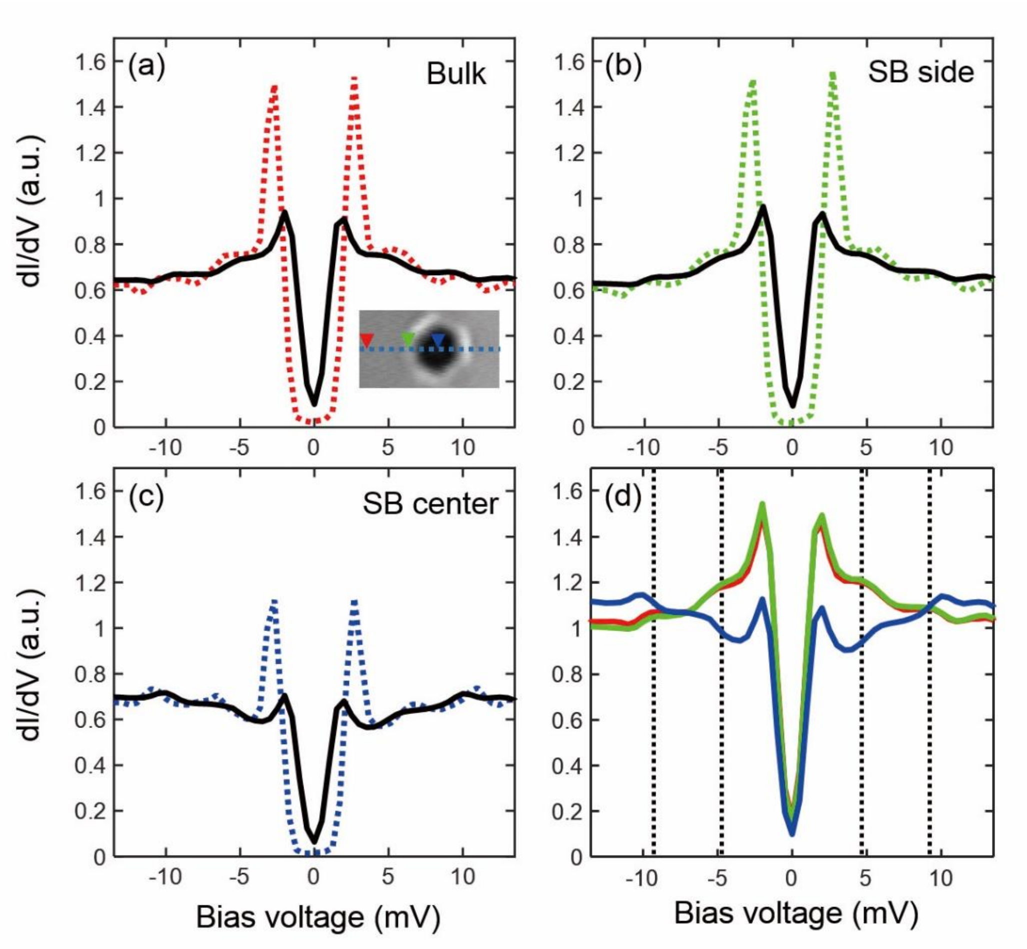

Figure S3. Deconvolution of the tunneling spectra measured by a superconducting tip. Using the reference spectrum measured by the normal tip, the deconvolution is performed for the spectra measured by a superconducting tip. The deconvolution algorithm of ref. 27 in main text is used. (a-c) The spectra measured by the superconducting tip (dotted lines) and spectra obtained by the deconvolution process (solid lines) at the Pb bulk, SB side and SB center. (d) Comparison among the spectra obtained by the deconvolution process. The vertical dotted line represents the position of phonon peaks at the Pb bulk and SB side. It is clear that the phonon peaks at the SB center are shifted outward in the spectrum.

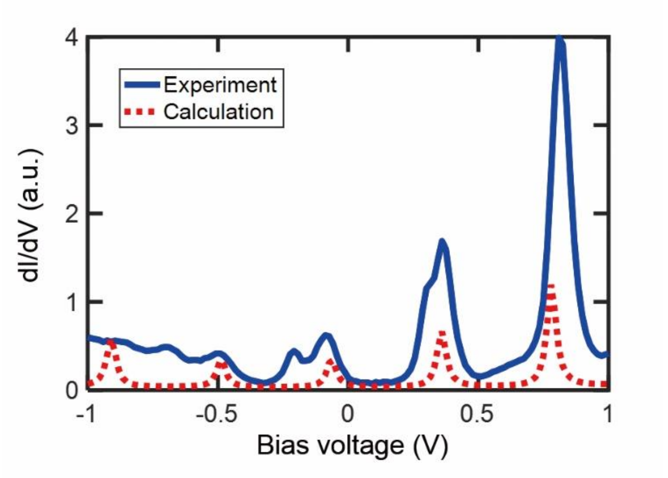

Figure S4. Determining of the depth of Ar-induced nano-cavities (AICs). The free standing Pb film is formed on AIC in Pb(111). We treat the Pb film as a Febry-Perot interferometer following the ref. S1. The Fabry-Perot spectral function for the 1-dimensional DOS of Pb film is given by $\rho \propto \frac{1}{1+\frac{4f^2}{\pi^2}\,sin^2\left(kNt+\frac{\emptyset}{2}\right)}$, where f is the interferometer finesse, k is the electron wavevector depending on energy, N is the number of monolayers (MLs), t is the thickness of a ML and Ø is a boundary phase shift. The blue solid line is the experimental data, and the red dotted line is calculated by the parameters of f = 20, N = 26 and Ø = 85.3.

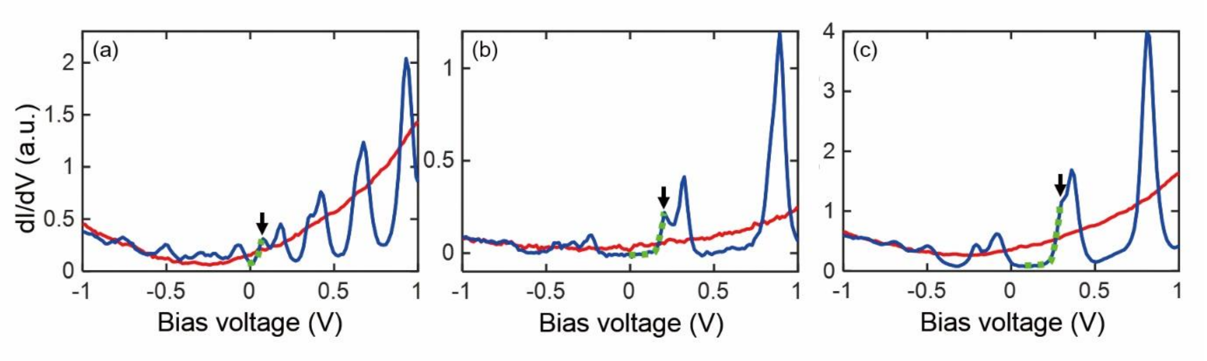

Figure S5. The position of the lowest unoccupied state (LU) and the density of states (DOS) at Fermi energy (E_F). (a–c) The dI/dV spectra at the center of three different SBs (blue lines) and bulk (red lines). The LU is located at 0.1 V, 0.2 V and 0.3 V, respectively (black arrow). The farther the LU from E_F, the less is the DOS at E_F affected by the broadened peak of LU (green dotted line). As a result, as the position of LU deviate from E_F, the DOS is decreased at E_F. Note that the highest occupied state is located away from E_F to minimize the system energy, thus barely affects the DOS at E_F.[S2] The rare exception we found in the experiment is SB A in Fig. 3 in main text.

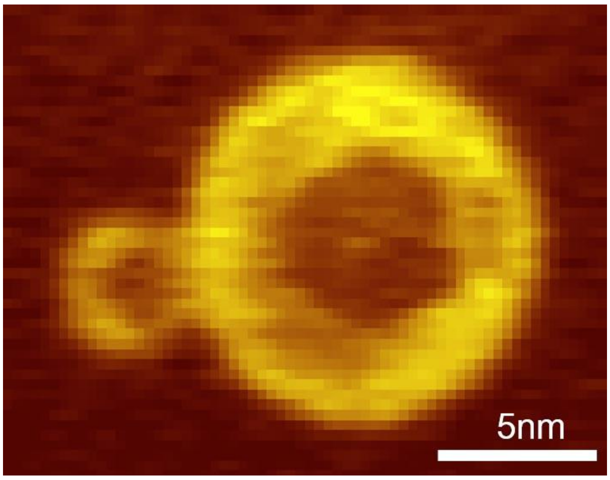

Figure S6. The size and symmetry of the enhanced superconducting gap near SBs. The conductance map at -2.7 mV is measured for two SBs. The area of the enhanced superconducting gap depends on the size of the AIC. The larger SB shows the larger area of the enhanced superconductivity. For both SBs, the enhanced superconducting region follows three-fold rotational symmetry.

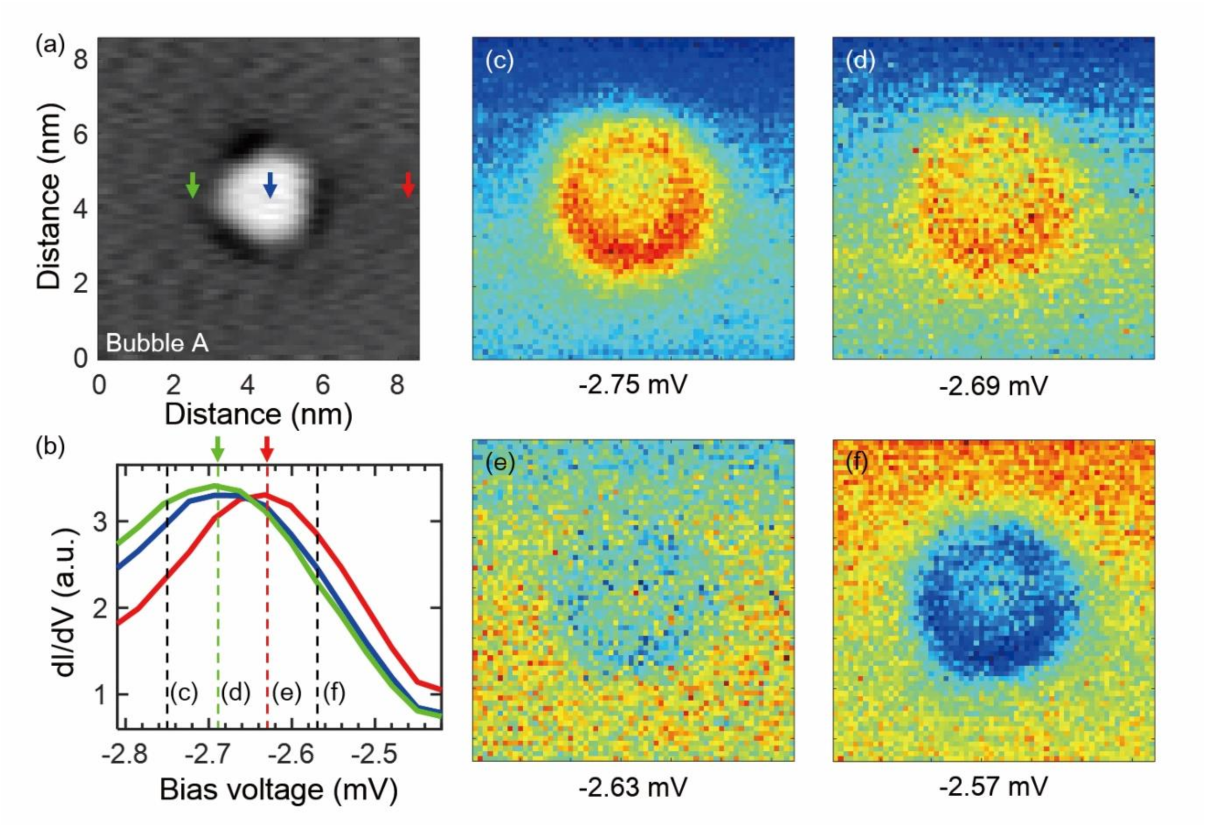

Figure S7. Differential conductance map depending on energy around an SB. (a) The topographic image of an SB of which diameter is 4 nm and depth is 34 ML. (V_{bias} = 0.05 V, I = 50 pA). (b) The quasiparticle resonance peaks at the SB side (green solid line), the SB center (blue solid line) and Pb bulk (red solid line). (c–f) The dI/dV map measured at the bias voltage of -2.75 mV, -2.69 mV, -2.63 mV, and -2.57 mV (see the vertical dotted lines in (b)). The bias voltage of -2.69 mV and -2.63 mV corresponds to the quasiparticle resonance peak measured at the SB side and Pb bulk, respectively.

REFERENCES

S1 Wang, K. D., Zhang, X. Q., Loy, M. M. T., Chiang, T. C. & Xiao, X. D. Pseudogap Mediated by Quantum-Size Effects in Lead Islands. *Phys Rev Lett* **102**, 076801 (2009).

S2 Li, S. C. *et al.* Influence of quantum size effects on Pb island growth and diffusion barrier oscillations. *Phys Rev B* **74**, 075410 (2006).
